# Effect of 3D laparoscopy versus traditional laparotomy on serum tumor markers and coagulation function in patients with early-stage endometrial cancer

**DOI:** 10.1016/j.clinsp.2024.100337

**Published:** 2024-02-17

**Authors:** Hailong Chen, Dechang Xu, Ying Yu, Jing Huang, Qian Zhou, Qi Wang

**Affiliations:** a Department of Gynecologic Oncology, Ganzhou Cancer Hospital, Ganzhou, Jiangxi, China; b Ganzhou Cancer Hospital, Ganzhou, Jiangxi, China

**Keywords:** Endometrial cancer, Early stage, 3D laparoscopic surgery, Laparotomy

## Abstract

**Objectives:**

To investigate the impact of Three-Dimensional (3D) laparoscopy compared to traditional laparotomy on serum tumor markers and coagulation function in patients diagnosed with early-stage Endometrial Cancer (EC).

**Method:**

The authors retrospectively analyzed the clinical data of 75 patients diagnosed with early-stage EC and categorized them into two groups based on the surgical techniques employed. The 3D group consisted of 36 patients who underwent 3D laparoscopic surgery, while the Laparotomy group comprised 39 patients who underwent traditional laparotomy. The authors then compared the alterations in serum tumor markers and coagulation function between the two groups.

**Results:**

Postoperatively, serum levels of CA125, CA199, and HE4 were notably reduced in both groups on the third day, with the levels being more diminished in the 3D group than in the Laparotomy Group (*p* < 0.05). Conversely, FIB levels escalated significantly in both groups on the third-day post-surgery, with a more pronounced increase in the 3D group. Additionally, PT and APTT durations were reduced and were more so in the 3D group than in the laparotomy group (*p* < 0.05).

**Conclusions:**

When juxtaposed with traditional laparotomy, 3D laparoscopic surgery for early-stage EC appears to be more efficacious, characterized by reduced complications, and expedited recovery. It can effectively mitigate serum tumor marker levels, attenuate the inflammatory response and damage to immune function, foster urinary function recovery, and enhance the quality of life. However, it exerts a more significant influence on the patient's coagulation parameters, necessitating meticulous prevention and treatment strategies for thromboembolic events in clinical settings.

## Highlights

• 3D laparoscopic surgery for early-stage EC seems to be more effective than traditional laparotomy.
• 3D laparoscopic surgery for early EC can attenuate the inflammatory response and damage to immune function, foster urinary function recovery, and enhance the quality of life.
• 3D laparoscopic surgery for early EC impacts the patient's coagulation function, necessitating vigilant prevention and management of thrombotic events in clinical settings.

## Introduction

Endometrial Cancer (EC) ranks as the third most prevalent gynecological malignancy in China, surpassed only by cervical and ovarian cancers. Alarmingly, the incidence of EC has surged in recent years, with a notable shift toward younger age groups, posing severe threats to patients' well-being and quality of life.^1^ Patients in the early stages of EC typically exhibit symptoms such as irregular vaginal discharge and bleeding. Due to heightened health awareness and advancements in medical technology, the majority of EC cases are now diagnosed early.^2^ Presently, surgical intervention is the primary treatment for early-stage EC. Most patients undergoing surgery experience therapeutic benefits, boasting a survival rate exceeding 80 % over a five-year span, and a minimal mortality rate.^3^^,^^4^ Historically, traditional laparotomy was the go-to approach for early EC, effectively removing the lesions. However, this method was associated with greater trauma, increased complications, and prolonged recovery.^5^ Thus, refining treatment strategies for early EC has become a pivotal research focus in leading medical institutions.

In light of ongoing medical advancements, laparoscopic surgery has gained traction for the clinical management of early-stage EC. This method is lauded for its minimal invasiveness, reduced intraoperative bleeding, and fewer complications, subsequently leading to a marked reduction in hospital stays.^6^^,^^7^ Yet, conventional laparoscopic procedures primarily offer two-dimensional visualizations, devoid of spatial depth and orientation. This limitation impedes the accurate portrayal of innate anatomical structures, compelling surgeons to rely heavily on their clinical expertise to decipher anatomical nuances, thereby amplifying operational challenges.^8^ In contrast, Three-Dimensional (3D) laparoscopic technology furnishes surgeons with accurate spatial and stereoscopic perspectives, engrossing them in a genuine three-dimensional operative environment. This immersion simplifies the procedure and enhances surgical outcomes.^9^ Notwithstanding its potential, there are scant clinical evaluations of 3D laparoscopic techniques in treating early-stage EC. To bridge this knowledge gap, the present study juxtaposed the impacts of 3D laparoscopy against traditional laparotomy, focusing on serum tumor markers and coagulation function in early-stage EC patients.

## Materials and methods

### Clinical data

The authors conducted a retrospective analysis of the clinical data from 75 early-stage EC patients treated at the studied hospital between March 2019 and June 2022. Their ages ranged from 37 to 65 years, with an average age of 53.86±5.77 years. According to FIGO staging, 34 patients were in stage I and 41 were in stage II. In terms of pathology, 64 patients had endometrioid adenocarcinoma, 8 had plasma adenocarcinoma, and 3 had clear-cell carcinoma. The body mass index ranged from 17 to 28 kg/m^2^, with a mean of 22.37 ± 1.84 kg/m^2^. Based on the ASA classification, there were 30 cases in Grade I and 45 in Grade II. Patients were categorized into two groups based on the surgical plan: the 3D group (n = 36) and the open surgery group (n = 39). This study was approved by the Ethics Committee of Ganzhou Cancer Hospital. The research objects were informed, and they signed a fully informed consent form.

### Inclusion and exclusion criteria

#### Inclusion criteria

Patients meeting the diagnostic criteria for EC,^10^ as confirmed by surgical pathology. Patients with comprehensive clinical data. Age range of 30‒70 years. FIGO stage I or II. Absence of contraindications to surgery. An anticipated survival time exceeding 6 months. ASA classification of Grade I or II.

#### Exclusion criteria

Pregnant or lactating women. Patients with distant tumor metastasis. Those diagnosed with other types of malignancies or possessing severe organ function abnormalities. Individuals with a prior history of uterine surgeries. Patients who had undergone radiotherapy before the study's initiation. Those diagnosed with severe internal diseases or psychiatric disorders.

## Methods

### Laparotomy group

The procedure commences with a conventional laparotomy. Sequentially: Anesthesia is administered via endotracheal intubation for general anesthesia. The patient is positioned supinely. A surgical incision, approximately 15 cm in length, is rendered along the midline of the lower abdomen. Upon accessing the abdominal cavity, a meticulous examination of the pelvic conditions is conducted.

The peritoneum is extended to the common iliac artery. Lymph nodes, including the common iliac, internal iliac, external iliac, obturator, and inguinal, are extracted, as is the round ligament. The pelvic infundibuliform ligament is ligated, adjacent uterine tissues are coagulated, and a segment of the vaginal wall is excised for pathological scrutiny. The residual vaginal end is meticulously sutured to preclude active bleeding.

Following the purification of the abdominal cavity, standard closure techniques are implemented.

### 3D group

This procedure employs three-dimensional laparoscopic surgery. Specifically: Anesthesia is induced through endotracheal intubation.

The patient assumes the lithotomy position. The medical team don's 3D glasses to afford a stereoscopic visualization of the pelvic region, facilitated by the three-dimensional laparoscopic system. Post-anesthesia, a uterine manipulator is positioned. An incision, approximately 1 cm in length, is made about 2 cm above the navel. The abdominal layers are accessed sequentially, and a 10 mm Trocar is situated to establish a CO_2_ pneumoperitoneum, with pressure sustained between 12‒15 mmHg.

A laparoscopic 10 mm Trocar is inserted just above the navel. Subsequent Trocars, of dimensions 10 mm, 5 mm, and 5 mm, are strategically placed at the left McBurney's point, the left rectus abdominis level with the navel, and the right McBurney's point, respectively. The abdominal and pelvic structures undergo a routine assessment.

Any prevalent adhesions are disentangled using either an ultrasonic scalpel or through blunt dissection. The left pelvic infundibuliform ligament is revealed up to the peritoneum and then sectioned. After opening the sheath of the left common iliac artery, the left internal iliac lymph nodes are excised. Lymph nodes, spanning both sides, including common iliac, internal iliac, external iliac, obturator, and inguinal, are extracted. The peritoneum overlaying the abdominal aorta is unsealed, facilitating the removal of some peri-aortic lymph nodes. The round ligament is bisected utilizing an ultrasonic scalpel. Exposure of the pelvic infundibuliform ligament is followed by bipolar coagulation and subsequent severance with an ultrasonic scalpel. Both the vesicouterine pouch and broad ligament are accessed, tissues proximal to the uterus are disassociated, and the uterine artery is exposed and cut. The uterus is meticulously extirpated along the periphery of the uterine manipulator. A comprehensive irrigation of the pelvic region is undertaken. The remaining vaginal end is secured with sutures, ensuring the cessation of any active bleeding. The abdominal cavity is then sanitized, concluding with standard closure procedures.

## Observation indicators

### Perioperative parameters

Evaluation of the two groups will involve comparing operation durations, intraoperative hemorrhage, lymph node dissections, time to the first post-operative flatus, catheter retention durations, and lengths of hospital stay.

### Tumor markers

From both groups, 3 mL of venous blood is collected on an empty stomach during the early morning, both pre-operatively and 3 days post-operatively. Following a 10 min centrifugation at 3000 rpm (radius of 6 cm), the supernatant serum is transferred to 1.5 mL EP tubes and stored at -80°C for subsequent analysis. Electrochemiluminescence will be employed to determine glycan antigen levels (CA125, CA199) and Human Epididymal secretion protein 4 (HE4). The kits are supplied by Beijing Kang Rui Na Biotechnology Co.

### Coagulation metrics

Indices such as Fibrinogen (FIB), Prothrombin Time (PT), and Activated Partial Thromboplastin Time (APTT) will be gauged using an automated hematology analyzer in both groups before and 3-days following the surgery.

### Immune profiling

The subsets of T-lymphocytes (CD3+, CD4+, CD8+, and CD4+/CD8+ ratios) are quantified by flow cytometry for both cohorts, both pre-surgery and 3 days post-surgery.

### Inflammatory markers

3 mL of venous blood, drawn from fasting subjects in the early morning, will be obtained from both groups before and 3 days after the procedure. Post a 10 min centrifugation (3000 rpm with a 6 cm radius), the resultant serum is secured in 1.5 mL EP tubes and refrigerated at -80°C until analysis. Levels of Tumor Necrosis Factor-alpha (TNF-alpha), Interleukin-6 (IL-6), and Procalcitonin (PCT) will be assayed using the ELISA technique. The diagnostic kits are courtesy of Bohui Biotechnology (Guangzhou) Co.

### Urinary function assessment

Both groups will undergo evaluations of residual urine volume, peak urinary flow, average urinary flow, and maximal urethral pressure using urodynamic measurements, conducted both pre-surgery and 3 months post-surgery.

### Quality-of-life analysis

The well-being of subjects from both groups will be assessed by employing the Quality-of-Life Assessment Scale for Cancer Patients (QLQ-30).^11^ This evaluation, conducted both pre-surgery and 3 months post-surgery, encompasses four dimensions: social relationships, physiological functionality, psychological health, and environmental factors. Each dimension carries a maximum score of 100, with diminished scores indicative of poorer quality-of-life.

### Post-operative complications

These may comprise urinary retention, wound infections, lymphocele formation, and intestinal obstructions, among others.

### Statistical analysis

The data was processed and analyzed using SPSS version 24.0 software. Continuous variables such as perioperative parameters, serum tumor markers, coagulation metrics, immune profiling, inflammatory markers, urinary function, and quality of life metrics were presented as mean ± Standard Deviation (χ±s). For comparing between groups, the independent samples *t*-test was employed, while within-group comparisons utilized the paired samples *t*-test. Categorical data were represented as frequencies (n) with percentages (%) and were compared using the χ^2^ test. A p-value less than 0.05 (*p* < 0.05) was deemed statistically significant.

## Results

### Clinical data comparison between the laparotomy and 3D groups

There was no statistically significant difference between the two groups in terms of age, FIGO stage, pathological type, body mass index, and ASA classification (p > 0.05). Refer to Table 1 for details.

**Table 1:** Comparison of clinical data n/(χ±S).

Group	Age (years)	FIGO Staging		Pathology type			Body mass index (kg/m^2^)	ASA grading	
		Stage I	Stage II	Endometrioid adenocarcinoma	Plasma adenocarcinoma	Clear cell carcinoma		Grade I	Class II
Laparotomy group (n = 39)	53.72 ± 6.15	18	21	34	4	1	22.49 ± 2.06	17	22
3D Group (n = 36)	54.08 ± 6.37	16	20	30	4	2	22.21 ± 1.97	13	23
*t*/χ^2^	0.249	0.022		0.197			0.601	0.436	
p	0.804	0.882		0.844			0.550	0.509	

### Perioperative indexes comparison between the laparotomy and 3D groups

The 3D group demonstrated reduced intraoperative bleeding, earlier time to first flatus, shorter catheter retention, and reduced hospitalization duration compared to the laparotomy group (*p* < 0.05). However, the operation time and the number of lymph nodes dissected showed no significant differences between the two groups (p > 0.05). Further information can be found in Table 2.

**Table 2:** Comparison of perioperative indexes (χ±S).

Group	Operation time (min)	Intraoperative bleeding volume (mL)	Number of lymphatic dissection (pcs)	Time to first flatus (h)	Catheter indwelling time (d)	Length of hospitalization (d)
Laparotomy group (n = 39)	185.69 ± 22.85	258.65 ± 18.23	19.58 ± 2.14	37.89 ± 2.92	2.79 ± 0.86	11.75 ± 2.15
3D Group (n = 36)	187.45 ± 19.74	182.41 ± 20.05	20.01 ± 2.02	30.57 ± 3.14	1.83 ± 1.02	8.43 ± 1.67
*t*	0.356	17.249	0.893	10.461	4.418	7.424
p	0.723	< 0.001	0.375	< 0.001	< *p* < 0.001	0.375 < 0.001

### Tumor marker levels comparison between the laparotomy and 3D groups

**Fig. 1:**
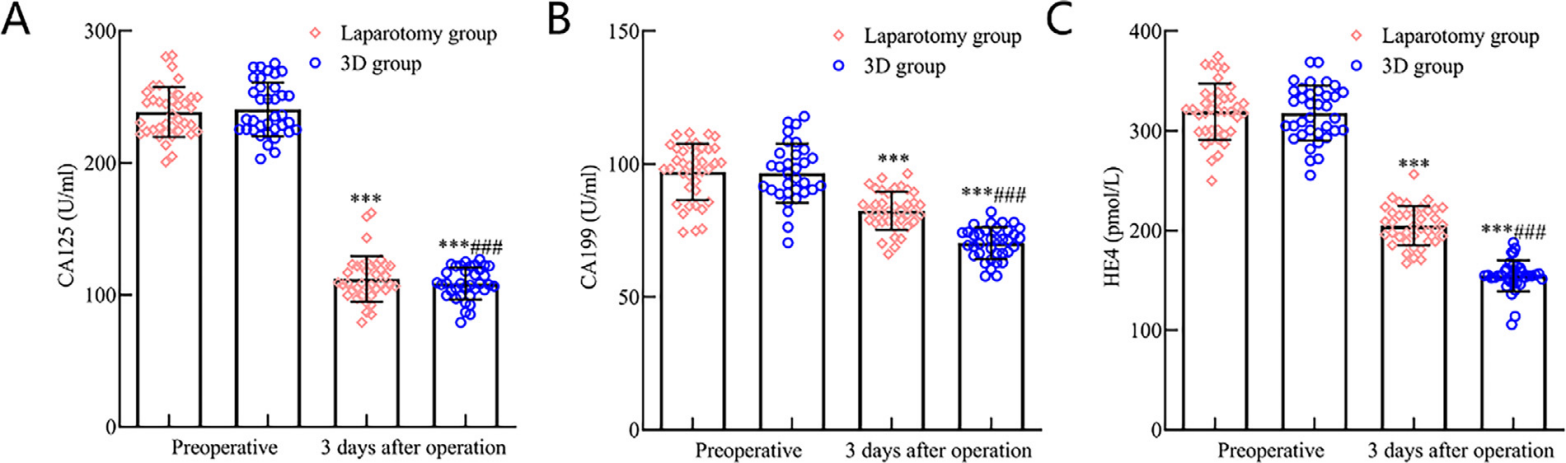
Comparative analysis of tumor marker levels in early-stage endometrial cancer patients following 3D laparoscopy and traditional laparotomy. Fig. 1 illustrates the postoperative serum levels of (A) CA125, (B) CA199, and (C) HE4 tumor markers. Notably, three days post-surgery, there was a pronounced reduction in the levels of all these markers in both the 3D laparoscopic group and the laparotomy group. Nevertheless, the decrease was more prominent in the 3D laparoscopic group compared to the laparotomy group (*p* < 0.05). Note: Compared with the preoperative period, *** *p* < 0.001; compared with the laparotomy group, ### *p* < 0.001.

There were no significant differences in preoperative levels of CA125, CA199, and HE4 between the two groups (p > 0.05). Postoperatively at 3 days, both groups exhibited a decline in CA125, CA199, and HE4 levels; however, these levels were more pronouncedly decreased in the 3D group compared to the laparotomy group (*p* < 0.05). See Fig. 1 for a visual representation.

#### Coagulation function comparison between the laparotomy and 3D groups

**Fig. 2:**
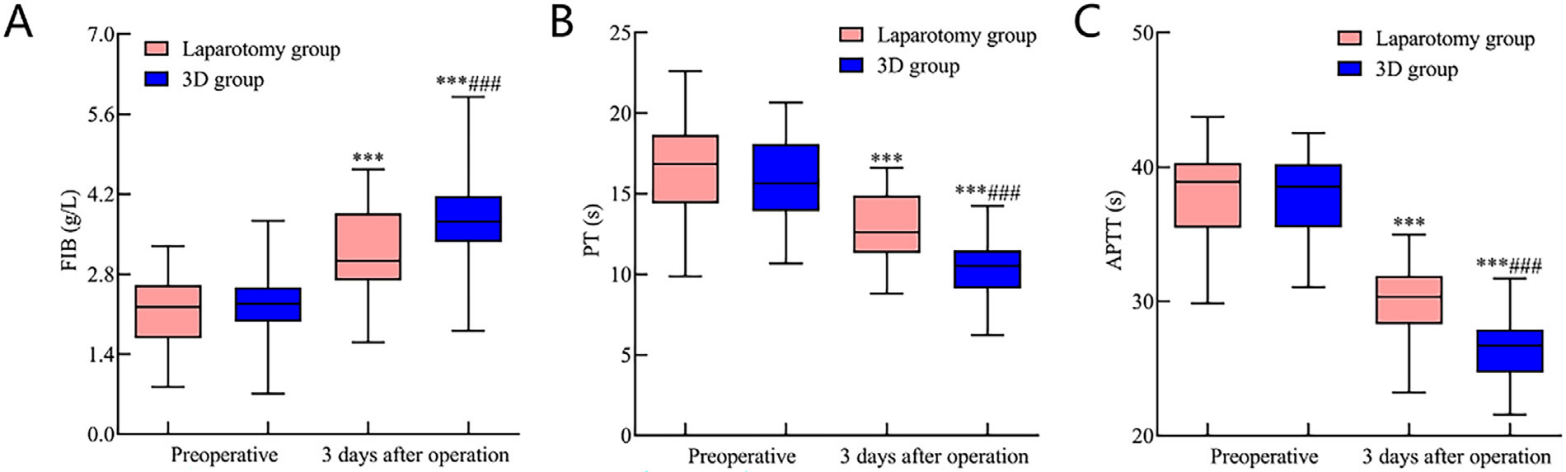
Comparative assessment of coagulation function indicators in early endometrial cancer patients following 3D laparoscopy and traditional laparotomy. Fig. 2 delineates the postoperative status of coagulation function indicators three days after surgery. Specifically, (A) FIB levels surged in both groups, with a more pronounced elevation in the 3D laparoscopic group compared to the laparotomy group. Conversely, durations for both (B) PT and (C) APTT diminished, with these reductions being more marked in the 3D laparoscopic group than in the laparotomy group. Note: Compared with the preoperative period, ^⁎⁎^* p < 0.001; compared with the laparotomy group, ^###^*p* < 0.001.

No statistically significant differences were observed in the preoperative levels of FIB, PT, and APTT between the two groups (p > 0.05). At 3 days post-operation, the FIB level in both groups rose, being notably higher in the 3D group than in the laparotomy group. Conversely, PT and APTT durations were reduced, and these reductions were more significant in the 3D group (*p* < 0.05). Refer to Fig. 2 for details.

### Immune function comparison between the laparotomy and 3D groups

**Fig. 3:**
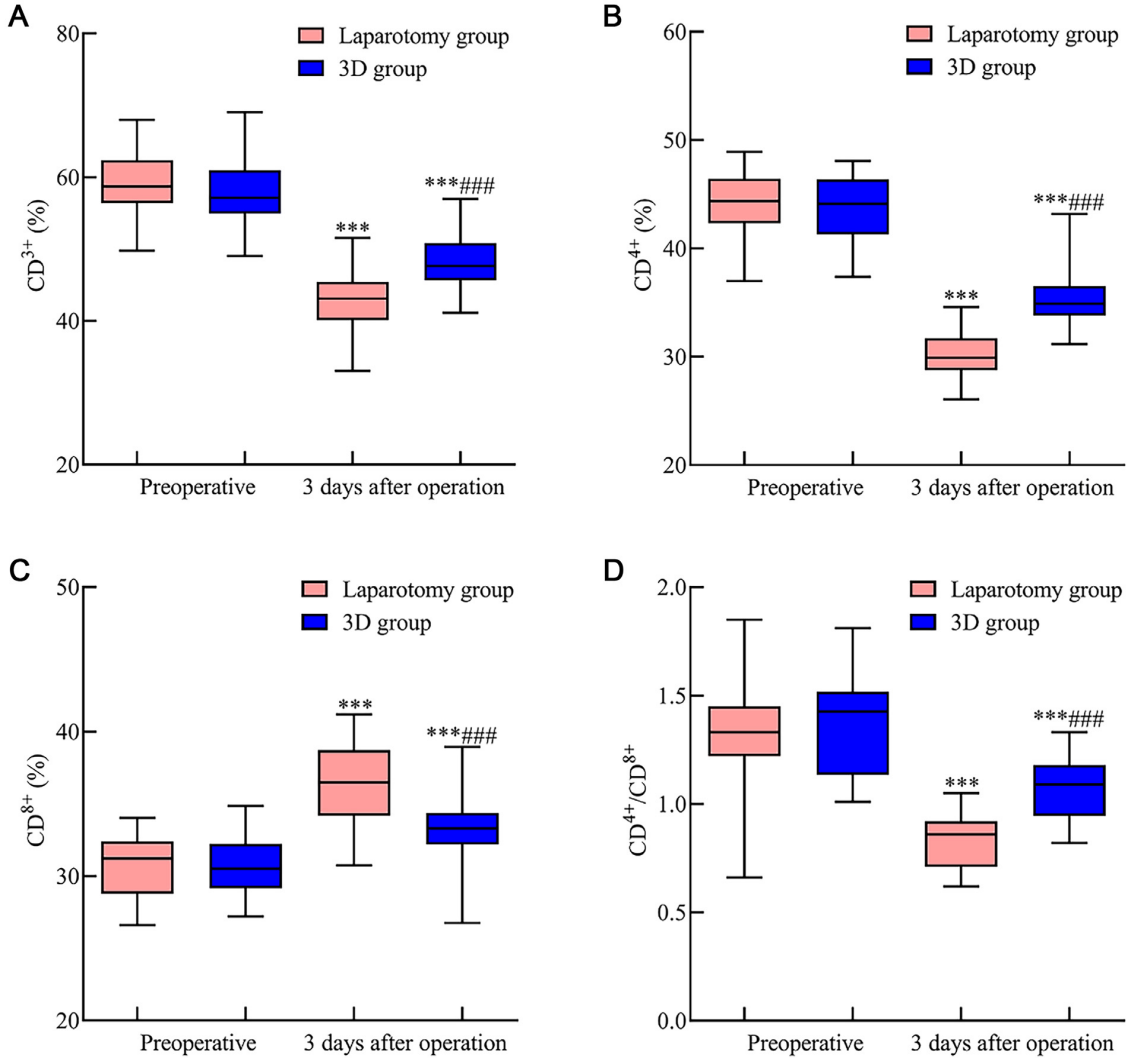
Comparative evaluation of immune function indicators in early endometrial cancer patients following 3D laparoscopy and traditional laparotomy. Fig. 3 illustrates the postoperative changes in immune function indicators three days after surgery. Specifically: (A) CD3+ levels decreased in both surgical groups yet remained relatively higher in the 3D laparoscopic group compared to the laparotomy group. (B) CD4+ levels followed a similar trend, with the 3D laparoscopic group showing a lesser decline than the laparotomy group. (C) CD8+ levels, on the other hand, exhibited an increase postoperatively in both groups. Notably, this increase was more pronounced in the 3D laparoscopic group than in the laparotomy group. (D) The CD4+/CD8+ ratio also declined post-surgery, but this decline was less significant in the 3D laparoscopic group when compared to the laparotomy group. Note: Compared with the preoperative period, *p* < 0.001; compared with the laparotomy group, ^###^*p* < 0.001.

Comparing the preoperative levels of CD3+, CD4+, CD4+/CD8+, and CD8+, no significant disparities were identified between the groups (p > 0.05). At 3-days post-surgery, both groups presented a decrease in CD3+, CD4+, and CD4+/CD8+ levels, but these levels remained higher in the 3D group. In contrast, CD8+ levels increased in both groups but were less elevated in the 3D group (*p* < 0.05). These variations are illustrated in Fig. 3.

### Inflammatory response comparison between the laparotomy and 3D groups

**Fig. 4:**
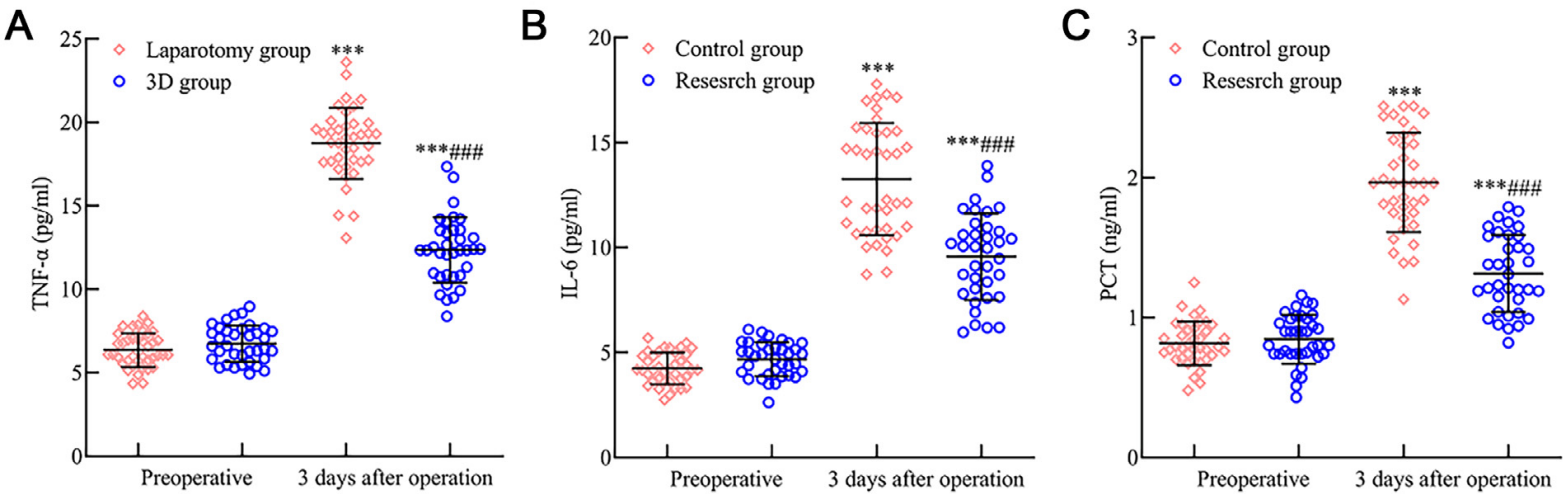
Comparative analysis of inflammatory response indicators in early endometrial cancer patients after 3D laparoscopy and traditional laparotomy. Fig. 4 delineates the variation in postoperative inflammatory response markers: (A) TNF-α levels surged in both surgical cohorts three days after surgery but exhibited a more subdued rise in the 3D laparoscopic group compared to the laparotomy group. (B) IL-6 and (C) PCT followed a similar trend, presenting heightened levels, with the 3D laparoscopic group showing relatively lower increments than the laparotomy group. Note: Compared with the preoperative period, *p* < 0.001; compared with the laparotomy group, ^###^*p* < 0.001.

There was no significant difference between the two groups in preoperative levels of TNF-α, IL-6, and PCT (p > 0.05). However, postoperative day 3 saw an elevation in TNF-α, IL-6, and PCT levels in both groups, with the 3D group having lower levels than the laparotomy group (*p* < 0.05). This data is depicted in Fig. 4.

### Urinary function comparison between the laparotomy and 3D groups

**Fig. 5:**
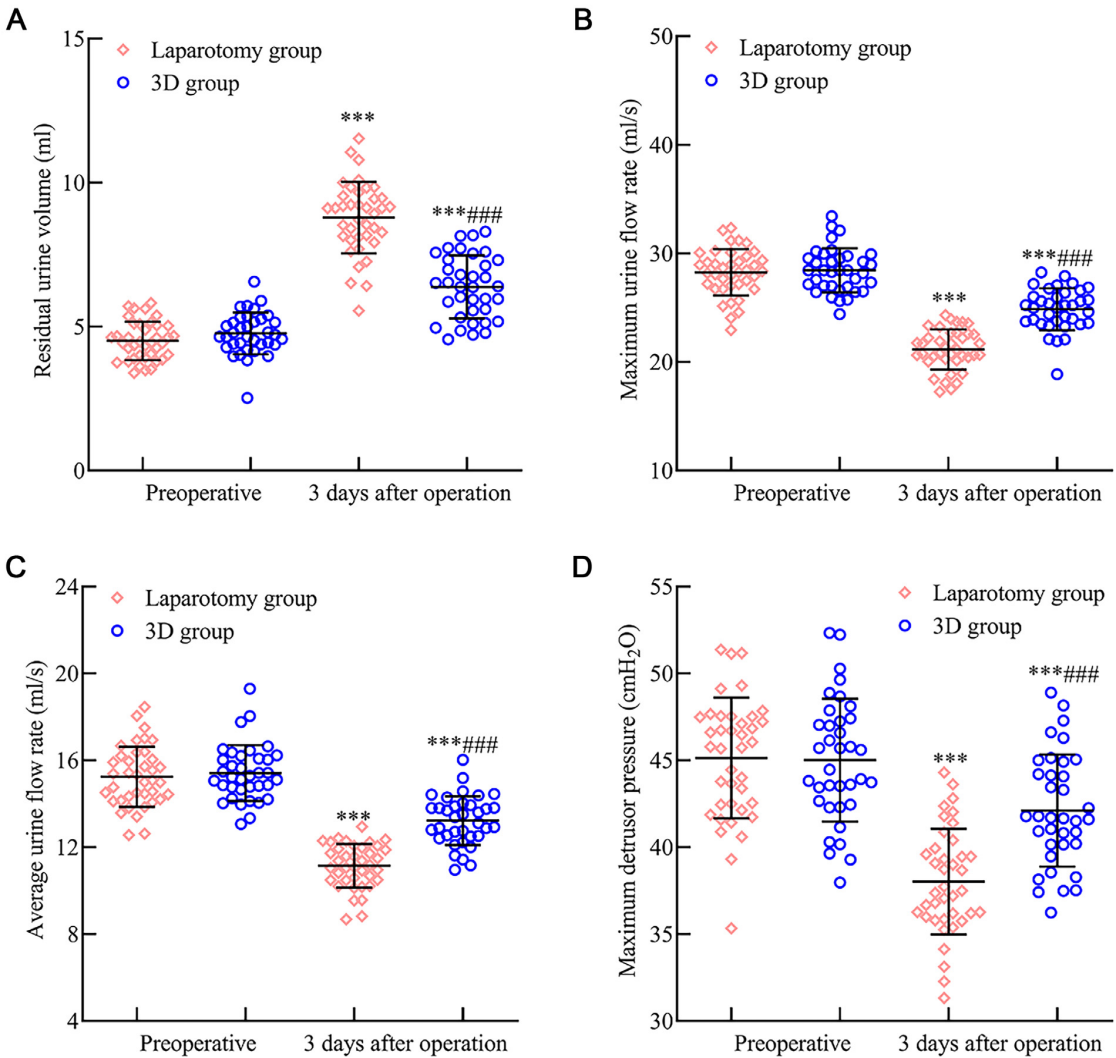
Assessment of inflammatory response indicators post 3D laparoscopic and traditional laparotomy in early endometrial cancer patients. Three months post-surgery, Fig. 5 reveals: (A) A marked increase in residual urine volume in both groups. However, this increase was less pronounced in the 3D laparoscopic group. (B) The maximum urinary flow rate and (C) The average urinary flow rate were discernibly reduced, yet these reductions were less substantial in the 3D laparoscopic group. (D) Conversely, the maximal pressure exerted by the forced urethral muscle was elevated, with the 3D laparoscopic group exhibiting a greater increase than the laparotomy group. Note: Compared with the preoperative period, *p* < 0.001; compared with the laparotomy group, ^###^*p* < 0.001.

No significant differences in preoperative urinary function indicators were observed between the two groups (p > 0.05). Three months post-surgery, both groups manifested an increase in residual urine volume, with the 3D group showing a lesser increase than the laparotomy group. Meanwhile, maximal urinary flow rate, average urinary flow rate, and maximum urethral pressure displayed decreases, but these decreases were less substantial in the 3D group (*p* < 0.05). Refer to Fig. 5 for insights.

### Quality of life comparison between the laparotomy and 3D groups

**Fig. 6:**
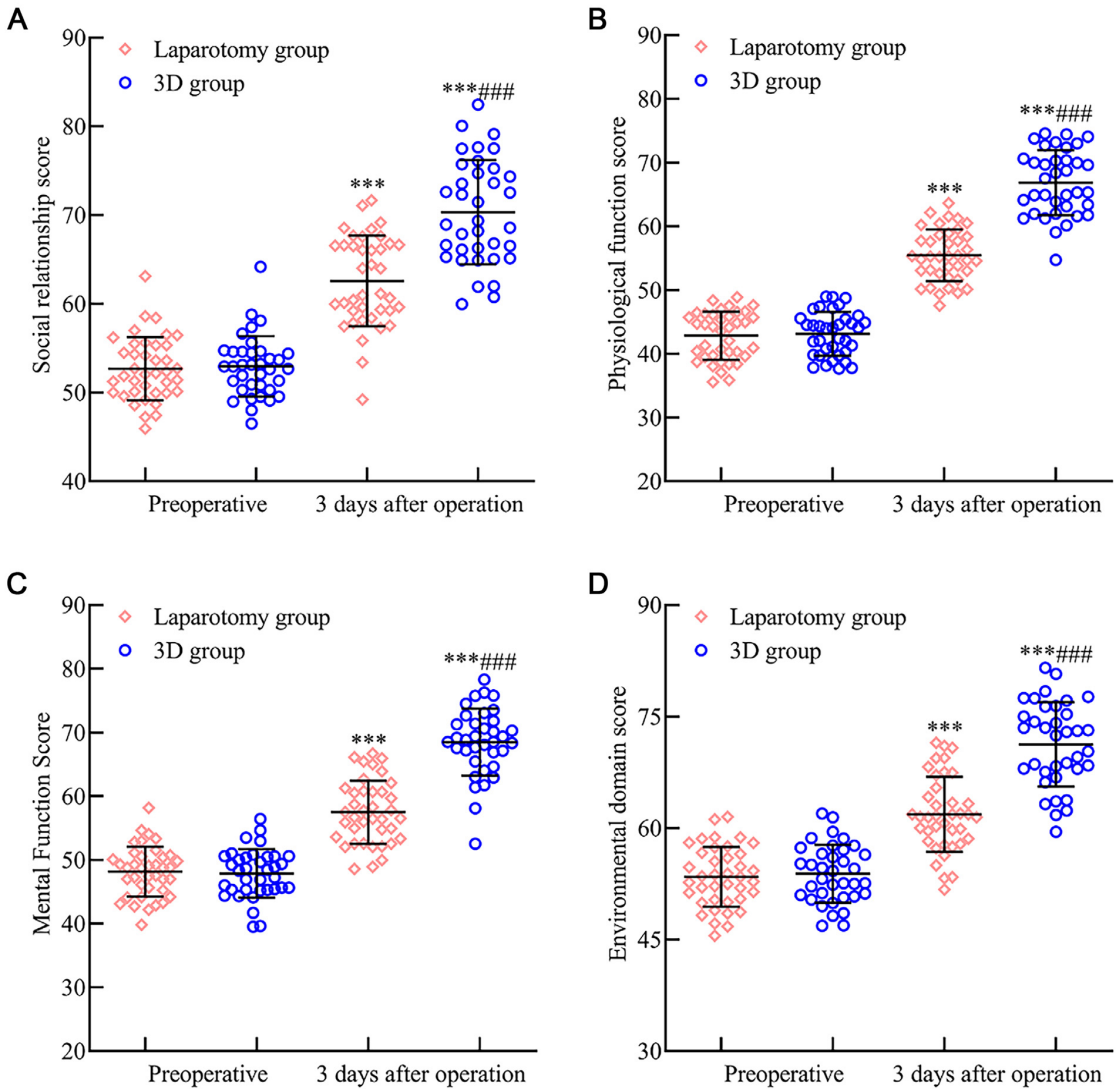
Evaluating the impact of 3D laparoscopy and traditional laparotomy on quality-of-life metrics in early endometrial cancer patients. Fig. 6 provides insights into the QLQ-30 scores for various domains three months post-surgery: (A) The social relationship score, (B) Physical function score, (C) Psychological function score, and (D) Environmental domain score all experienced a notable enhancement in both surgical groups. Crucially, each of these scores was markedly superior in the 3D laparoscopic group compared to the laparotomy group. Note: Compared with the preoperative period, *p* < 0.001; compared with the laparotomy group, ^###^*p* < 0.001.

Comparative assessment of preoperative quality of life scores between the two groups revealed no significant difference (p > 0.05). Nonetheless, at 3 months post-operation, both groups recorded increased QLQ-30 index scores, with the scores being notably higher in the 3D group (*p* < 0.05). Further details are illustrated in Fig. 6.

### Complication rates comparison between the laparotomy and 3D groups

The 3D group exhibited a lower complication rate of 5.56 %, in contrast to 25.64 % in the laparotomy group (*p* < 0.05). Comprehensive data is presented in Table 3.

**Table 3:** Comparison of complications n (%).

Group	Urinary retention	Incision infection	Lymphocysts	Intestinal obstruction	Total
Laparotomy group (n = 39)	3 (7.69)	3 (7.69)	3 (7.69)	1 (2.56)	10 (25.64)
3D Group (n = 36)	1 (2.78)	1 (2.78)	0	0	2 (5.56)
χ^2^					5.619
p					0.018

## Discussion

Endometrial Cancer (EC) stands as a prevalent malignancy in gynecology, predominantly afflicting postmenopausal senior women. The etiology of EC is multifaceted, and predominantly linked to hypertension, cardiovascular diseases, genetic predisposition, obesity, and prolonged estrogen exposure.^12^ In contemporary medical practices, early-stage EC patients are primarily chosen for surgical interventions, which demonstrate significant efficacy.^13^ However, traditional laparotomy often subjects patients to pronounced trauma and increased postoperative complications, adversely affecting their prognosis.^14^ Though laparoscopic surgery promises reduced patient trauma and fewer postoperative complications, it suffers from a limited spatial orientation and depth perception, rendering it challenging.^15^^,^^16^

In the present research, the authors integrated 3D laparoscopic techniques for patients with early EC. Data indicates that the 3D cohort experienced reduced intraoperative bleeding, lesser anal exhaustion, shorter catheter retention and hospital stays, and a diminished complication rate (5.56 %) as opposed to the laparotomy group's 25.64 %. Furthermore, voiding function and quality of life, assessed three months post-surgery, were superior in the 3D group (*p* < 0.05). This suggests that for early-stage EC, 3D laparoscopy, in contrast to traditional laparotomy, offers precision, fewer complications, expedited recovery, and supplementary benefits. These can actively bolster urinary function restoration and elevate life quality. Factors contributing to these benefits include 3D laparoscopy's ability to offer surgeons a three-dimensional viewpoint, enhancing tissue structure differentiation and meticulous operation, Facilitating direct lesion site identification, allowing comprehensive cancerous tissue and metastatic lymphocyte removal, and Assisting precise adhesion differentiation between the lesion and adjacent tissues, preventing cancer cell proliferation and limiting damage to surrounding structures.^17^^,^^18^

Tumor markers, notably CA125, CA199, and HE4, play a pivotal role in EC's pathological trajectory.^19^ Notably, CA125, an endometrial-secreted glycoprotein antigen, stands out as a primary EC diagnostic tool. Investigations by Kakimoto S et al.^20^ revealed markedly elevated serum CA125 levels in EC patients compared to a healthy control group. CA199, a tumor-affiliated antigen, correlates with EC's invasive potential and mirrors patient prognosis. Meanwhile, HE4, an expressed glycoprotein in ovarian and endometrial cancers, further underscores its significance. The present findings highlight that postoperative 3D group patients had significantly reduced serum CA125, CA199, and HE4 levels compared to their laparotomy counterparts (*p* < 0.05). This might be attributed to 3D laparoscopy's precision and comprehensive lesion excision capabilities.

Surgical trauma can compromise immune function, disturb coagulation mechanisms, and escalate risks like secondary infections.^21^ Studies, including those by Qian Junxiong,^22^ observed hypercoagulability in malignancy patients. Herein, FIB offers insights into coagulation status, while PT and APTT shifts correlate with coagulation factor concentrations. Surgical stress responses can suppress both humoral and cellular immunity and catalyze inflammatory agents like TNF-α, IL-6, and PCT, influencing post-surgical recuperation.^23^^,^^24^ These observations underscore that, compared to traditional laparotomy, 3D laparoscopic surgery could effectively mitigate inflammatory and immunological repercussions in early EC patients, albeit influencing coagulation more. This discrepancy may arise from 3D laparoscopy-induced hypercoagulability due to pneumoperitoneum establishment, venous stasis from specific positioning, and the activation of the fibrinolytic system.^25^

However, during the surgical procedure, the authors encountered several challenges related to the 3D technology. Firstly, for those wearing 3D glasses for the first time, there was an initial discomfort and a required focus adjustment. This transition often led to eye strain and necessitated an acclimatization period. Secondly, the frequent transition between 3D stereoscopic vision and 2D planar vision during the operation resulted in visual fatigue, soreness, and other discomforts, such as eye irritation, especially after extended use of 3D glasses. Lastly, beginners to the 3D laparoscopic technique often perceive an exaggerated depth in the surgical field. This could easily lead to a misjudgment of the operative field's depth, necessitating a brief adaptation period.

## Conclusion

In conclusion, when juxtaposed with traditional laparotomy, 3D laparoscopic surgery for early EC exhibits remarkable precision. It offers distinct advantages, such as reduced complications, expedited recovery, effective reduction in serum tumor marker levels, decreased inflammatory responses, minimized immune function damage, enhanced urinary function recovery, and improved quality of life. However, it notably impacts the patient's coagulation function, necessitating vigilant prevention and management of thrombotic events in clinical settings. Yet, it's pertinent to note that the present study is characterized by its small sample size and its retrospective, single-center nature. These factors might introduce potential biases. Moreover, the present research did not delve into the long-term recurrence and prognosis of early EC patients’ post-3D laparoscopic surgery. Thus, there is an eager anticipation for extensive, prospective research in the future to provide a more comprehensive understanding.

## Funding

This research did not receive any specific grant from funding agencies in the public, commercial, or not-for-profit sectors.

## CRediT authorship contribution statement

**Hailong Chen:** Visualization, Writing – original draft. **Dechang Xu:** Visualization, Writing – original draft. **Ying Yu:** Formal analysis, Data curation. **Jing Huang:** Formal analysis, Data curation. **Qian Zhou:** Formal analysis, Data curation. **Qi Wang:** Visualization, Writing – review & editing.

## Declaration of competing interest

The authors declare no conflicts of interest.
